# The Morphology of Corneal Cap and Its Relation to Refractive Outcomes in Femtosecond Laser Small Incision Lenticule Extraction (SMILE) with Anterior Segment Optical Coherence Tomography Observation

**DOI:** 10.1371/journal.pone.0070208

**Published:** 2013-08-05

**Authors:** Jing Zhao, Peijun Yao, Meiyan Li, Zhi Chen, Yang Shen, Zhennan Zhao, Zimei Zhou, Xingtao Zhou

**Affiliations:** 1 Department of Ophthalmology, Eye and ENT Hospital of Fudan University, Myopia Key Laboratory of the Health Ministry, Shanghai, China; 2 Department of Ophthalmology, Bronx Lebanon Hospital Center, Albert Einstein College of Medicine, Bronx, New York, United States of America; Medical University Graz, Austria

## Abstract

**Purpose:**

To investigate the morphology of corneal caps in femtosecond laser small incision lenticule extraction (SMILE) and its relation to the refractive outcomes.

**Methods:**

A prospective study of fifty-four corneal caps created with VisuMax femtosecond laser were examined using an Fourier-domain optical coherence tomography at 1 day, 1 week, 1 month and 6 months after SMILE. The cap thickness at nine points on each of the four meridians (0°, 45°, 90°, 135°) and the diameter were measured. Cap morphology, changes over time and its correlation with refractive outcomes were assessed.

**Results:**

The mean achieved central cap thickness were (108.74±5.06) µm at 6 months and (107.32±4.81 ) µm at 1 month postoperatively, significantly thinner than that at 1 day (110.81±7.95) µm and 1 week (109.58±7.48 ) µm (*P<*0.05). The mean diameter on 0° meridian was (7.61±0.07) mm, significantly larger than that on 90° meridian (7.57±0.06) mm (*P = *0.001). Cap morphology showed good regularity, except that the differences of points in two pairs were significant at 1 day postoperatively. The uniformity was consistent over time and the central cap thickness was thinner than those in the paracentral and peripheral areas. The refractive outcomes stabilized within 1 month. Uncorrected distance visual acuity (UDVA) was correlated to the central cap thickness at 1 day and 1 week (both *r_s_* = 0.33, p<0.05). The uniformity index was correlated with UDVA (*r_s_* = 0.34, p<0.05) and corrected distance visual acuity (*r_s_* = 0.32, p<0.05) at 1 week postoperatively.

**Conclusions:**

Corneal caps of SMILE are predictable with good reproducibility, regularity and uniformity. Cap morphology might have a mild effect on refractive outcomes in the early stage. Further study should focus on the impact on the visual quality.

## Introduction

In the past few years, the introduction of femtosecond laser (FS) technology has been a remarkable innovation in the field of corneal refractive surgery. A recent breakthrough of FS technology resulted in a novel “all-in-one” refractive procedure called refractive lenticule extraction (ReLEx), which can be performed in two different modalities. One is the femtosecond lenticule extraction (FLEx), which involves creating and lifting a hinged flap followed by manual removal of an intrastromal refractive lenticule, a procedure very similar to laser in situ keratomileusis (LASIK). The other one is the small incision lenticule extraction (SMILE), an alternative flapless procedure, whereby a flap is not made and lifted but the lenticule is separated and removed through a small incision, leaving an interface in the cornea and the upper arcade of the corneal tissue (equivalent to a flap) called “cap” [1]–[6].

The predictability of flap creation is a significant contributing factor to successful LASIK treatment. Several studies have reported that FS tends to create uniform flaps with high predictability and minimal variability in thickness and diameter, resulting in better astigmatic neutrality and induction of fewer higher order aberrations [7]–[9]. A recent study has focused on the flap and stromal bed thickness after ReLEx. [10] However, to the best of our knowledge, few studies have been conducted to evaluate the morphological characteristics of SMILE corneal caps as well as their relation to the refractive outcomes. Although SMILE has been proved to be safe, predictable, and effective in treating myopia and myopic astigmatism in earlier clinical reports [1], [3], the qualitative aspect of corneal caps created in surgery remains largely unknown and is therefore of great interest. In this regard, it’s of significant value to characterize the morphology of SMILE corneal caps.

Fourier-domain optical coherence tomography (OCT) is useful in analyzing flap morphology due to its advantageous non-contact technique, relative ease of use, the ability to visualize wide areas of the cornea including multiple flap interfaces, and to directly measure flap thickness in different meridians [7], [9]. Moreover, these instruments can image the cornea with higher resolution and faster speed than time-domain OCT instruments of older generations [11], [12]. Thus, the present prospective study utilized anterior segment Fourier-domain OCT to perform non-contact comprehensive examination for corneal caps after SMILE. The predictability, regularity and uniformity of the caps created with 500 kHz VisuMax FS were assessed.

## Materials and Methods

### Ethics Statement

The study was performed in accordance with the Declaration of Helsinki and was reviewed and approved by the Ethics Committee of the Eye and ENT Hospital of Fudan University. Written informed consent was obtained from each patient after the nature and possible consequences of the study were explained.

### Patients

This prospective, non-randomized study included 54 eyes of 30 myopes (11 male, 19 female) with a mean age of 29.6±5.8 years (range, 22 to 44 years). The subjects were recruited from the Refractive Surgery Centre of the Department of Ophthalmology, Eye and ENT Hospital of Fudan University (Shanghai, China) between November 2011 and January 2012. Criteria for inclusion were: spherical refraction from −3 to −10 D, astigmatism less than 5 D, corrected distance visual acuity (CDVA) of 20/25 or better, stable refraction for two years prior to surgery and no use of any kind of contact lenses within the last two weeks. Exclusion criteria were suspicion of keratectasia, active ocular disease and history of prior ocular surgery or trauma. Routine preoperative examinations were conducted in each patient to rule out contraindications for the SMILE procedure. The mean preoperative spherical equivalent (SE) of the eyes was −6.67±1.43 D. The mean preoperative corneal keratometry was 43.17±1.49 D and corneal thickness was 547.20±33.10 µm.

### Surgical Technique

All SMILE procedures were performed by the same surgeon (XT.Z.) using a VisuMax femtosecond laser system (Carl Zeiss Meditec AG) with a repetition rate of 500 kHz and a pulse energy of 130 nJ. A series of bubbles were created in a spiral fashion with a spot distance of 2×2 µm resulting in cleavage of the following four tissue planes: the posterior surface of the refractive lenticule, the lenticule border, the anterior surface of the refractive lenticule, and a single small 90-degree angled side-cut incision with a circumferential length of 3 to 4.5 mm in the superior position. In all cases, the intended anterior surface of the lenticule was 100 µm deep, a depth equivalent to the thickness of the cap (equivalent of ‘flap thickness’ in standard FLEx), and its intended diameter was 7.5 mm, 1 mm larger than the diameter of the refractive lenticule. After the FS cutting procedure was finished, the refractive lenticule of intrastromal corneal tissue was dissected and separated through the side-cut opening incision and finally manually removed. The exact surgical maneuver was described elsewhere [1], [3]. All corneal caps were created uneventfully and no intra-operative or postoperative complications were observed. Postoperatively, patients were instructed to instill fluorometholone 0.1%, levofloxacin, and artificial tears four times per day in the operated eye for 2 weeks. Evaluations were performed 1 day, 1 week, 1 month and 6 months after surgery including manifest refraction, uncorrected distance visual acuity (UDVA), CDVA, topography, slit-lamp examination and anterior segment Fourier-domain OCT.

### Anterior Segment Optical Coherence Tomography

All the operated eyes were measured with a Fourier-domain OCT system (RTVue, software version 6.2, Optovue, Inc.), which has a sampling rate of 26 000 axial scans per second. A wide-angle (long lens) adapter lens was used in this study, which has a scan width of 8.0 mm, an axial resolution of 5 µm and a transverse resolution of 15 µm [7]. All measurements and evaluations were performed by the same examiner (J.Z.), who was masked to the intended corneal cap thickness. The cornea was imaged with a 8.0 mm long line scan pattern along the 0°, 45°, 90°, and 135° meridian, respectively. Sixteen consecutive frames were acquired with line scan mode for each scan.

**Figure 1:**
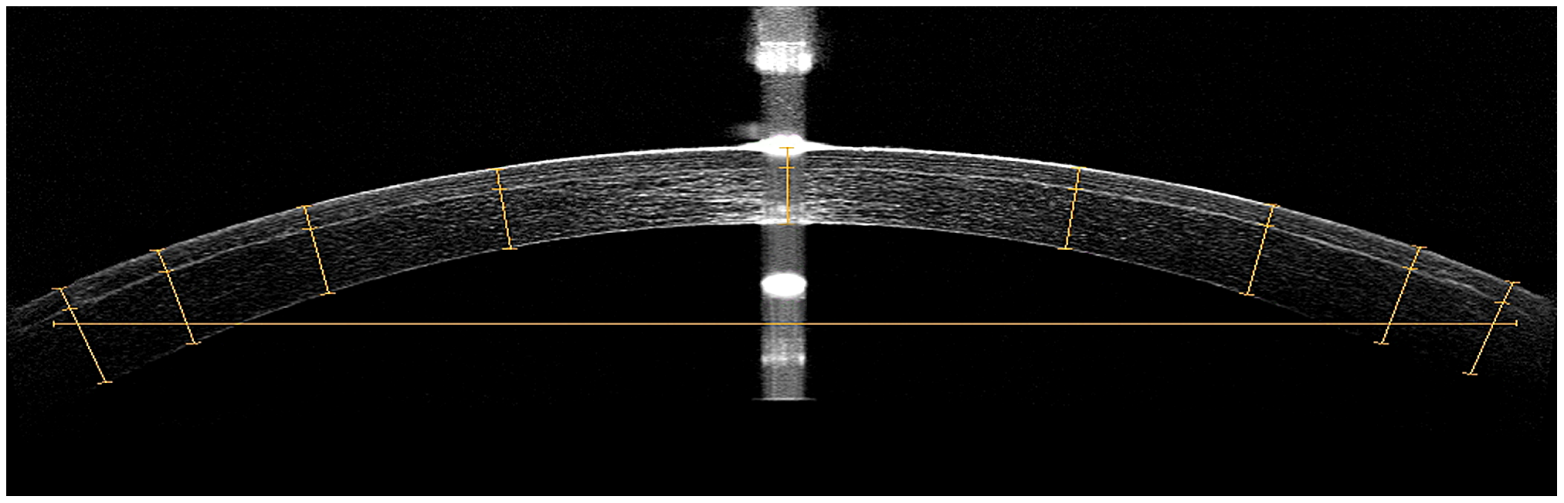
An example of a measurement image. Cap thickness was measured at the center, ±1.5 mm, ±2.5 mm, ±3.25 mm and ±3.75 mm from the center. Cap diameter was determined as the distance between the rims of the cap interface.

**Figure 2:**
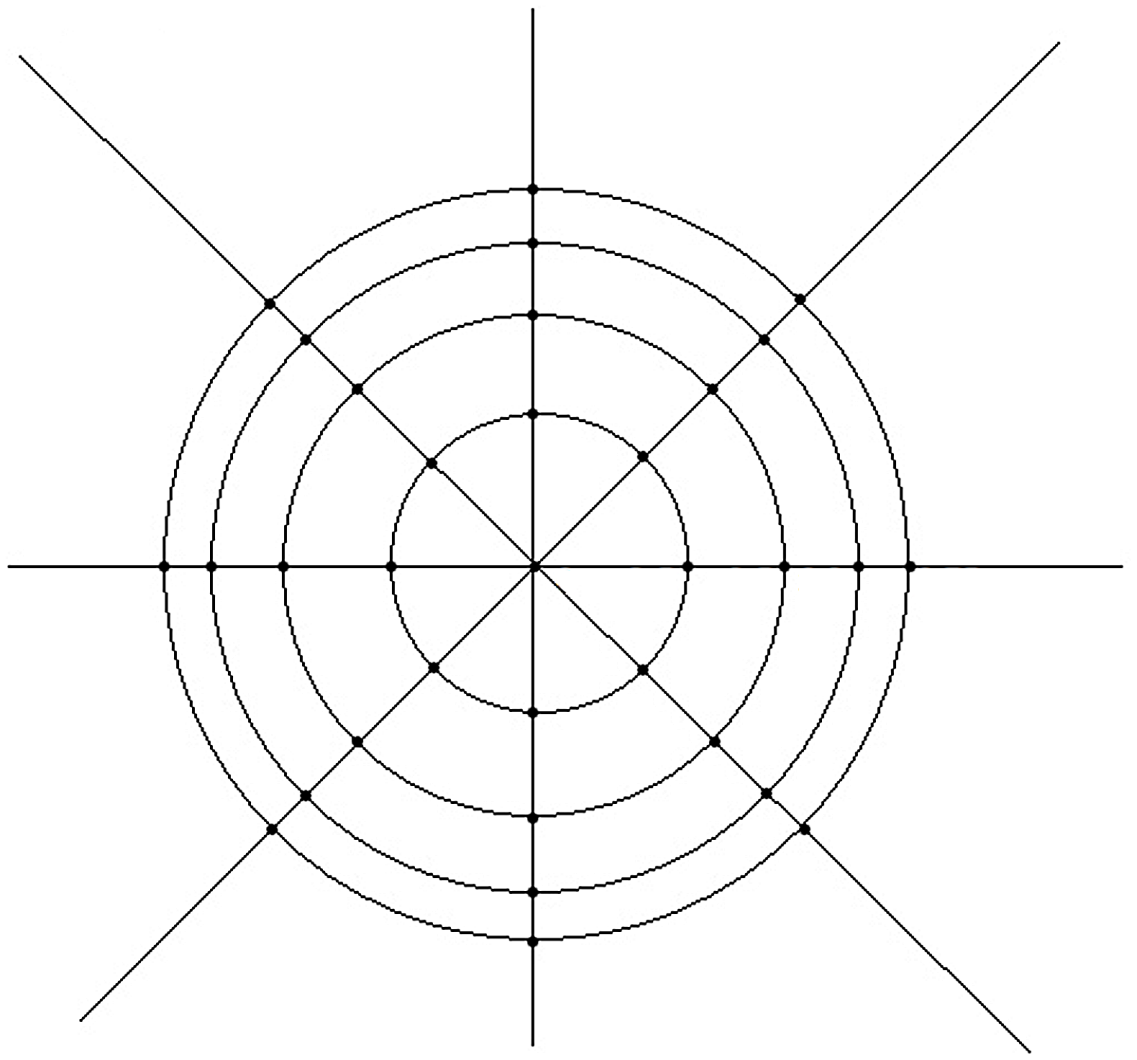
Top view of the measurement schematic of the whole cornea. Thirty-three points were analyzed for each corneal cap.

For line scans, the system’s software performs automatic registration to remove the corneal motion between the consecutive frames and provides an averaged image. More than one image was saved and reviewed and the image with the best centration and quality along each meridian was chose and evaluated by the examiner. The anterior and posterior corneal boundaries were acquired automatically and the interface was adjusted manually using calipers provided by the software. The corneal cap thicknesses at nine points (centre 0 mm, ±1.5 mm, ±2.5 mm, ±3.25 mm and ±3.75 mm from the centre) at the reflective line along each meridian were assessed at each visit. The corneal vertex is defined as 0 mm with negative on the left and positive on the right (Figure 1). Thus, each cap across the central 8 mm of the cornea was measured at 8 points of each eccentricity with a total of 33 points including the vertex (Figure 2). Cap diameter determined as the distance between the rims of the cap interface was also measured along the 0° and 90° meridian at 1 month postoperatively (Figure 1).

### Statistical Analyses

The accuracy of central corneal cap thickness and diameter was calculated as the difference between the achieved and intended values. Reproducibility was evaluated as the inter-individual standard deviation (SD) of the central cap thickness and diameter between eyes. Measurements of the diameter along the 0° and 90° meridians were further compared using a Paired *t* test. To evaluate the regularity of the caps, the mean cap thickness of each eight points at the same eccentricity were compared, respectively. The above mentioned values were compared at the different time-points using repeated-measures analysis of variance (ANOVA) and least squares difference analysis. Moreover, the cap measurements at the centre position along four medians were averaged to calculate the mean central cap thickness. Similarly, the cap measurements at the ±1.5 mm and ±2.5 mm positions were averaged to calculate the mean paracentral cap thickness while the ±3.25 mm and ±3.75 mm positions were averaged to calculate the mean peripheral thickness. To show the uniformity of the caps, a generalized linear mixed-model for repeated measures was applied to compare peripheral to central measurements.

Furthermore, the Spearman correlation analysis was used to assess the relationship between the postoperative refractive outcomes and the cap morphology. The parameters of refractive outcomes included SE, LogMAR UDVA, LogMAR CDVA and astigmatism. The parameters of cap morphology comprised of central cap thickness and the gross index of uniformity, *U*, defined as the standard deviation of the cap thickness of the total 33 points. The normality of the data distributions was tested using the Kolmogorov-Smirnov test. A *P* value of less than 0.05 was considered to be statistically significant. All data were collected using Excel software, version 2003 (Microsoft Corporation, Redmond, WA, USA). Statistical analyses were performed using SAS system software, version 8.2 (SAS Institute Inc., Cary, NC, USA).

## Results

### Cap Predictability

The mean achieved central cap thickness were 110.81±7.95 (range, 99.00 to 132.00) µm at 1 day, 109.58±7.48 (range, 98.50 to 126.00) µm at 1 week,107.32±4.81 (range, 100.00 to 118.25) µm at 1 month and 108.74±5.06 (range, 100.50 to 122.75) µm at postoperative 6 months, respectively. There were no significant changes in cap thickness between 1 day and 1 week as well as that between 1 month and 6 months after surgery. The central cap thickness after 1 month and 6 months were significantly thinner than that at 1 day and 1 week postoperatively (*P<*0.05). The mean achieved cap diameter on the 0° meridian was 7.61±0.07 (range, 7.51 to 7.83) mm, significantly larger than 7.57±0.06 (range, 7.46 to 7.68) mm on the 90° meridian (*P = *0.001) at 1 month postoperatively.

### Cap Regularity

Comparison of the cap thickness among the points in each eccentricity revealed good regularity over time. There was no statistically significant difference either among the central point along the four medians or among the 8 points in each eccentricity from the centre, except that the differences between two pairs of points along the 0° and 90° median in 1.5 mm and 2.5 mm eccentricities were statistically significant, only at 1 day postoperatively. (Table 1).

**Table 1:** Cap thickness (µm) at each measuring point over time (Mean ± SD).

Time	Location	0°	45°	90°	135°
**Post 1 day**	0 mm	110.23±9.02	109.28±10.34	111.77±8.40	112.00±9.01
	1.5 mm	113.21±9.22	113.51±10.54	115.09±9.51* *(p* = 0.0089)	114.12±9.99
	−1.5 mm	111.27±9.41*(*p* = 0.0089)	113.93±8.56	113.94±7.90	114.71±9.04
	2.5 mm	118.83±9.19 * (*p* = 0.046)	117.21±11.00	115.30±9.51* *(p* = 0.046)	115.83±10.20
	−2.5 mm	116.50±9.14	116.63±9.95	117.28±9.31	116.76±8.96
	3.25 mm	116.85±9.96	115.93±9.27	114.55±9.12	117.10±9.53
	−3.25 mm	115.42±9.75	116.72±8.27	115.49±7.95	116.38±7.43
	3.75 mm	115.65±10.25	115.02±10.00	113.26±8.23	115.31±9.93
	−3.75 mm	115.46±10.35	116.44±10.08	114.17±7.26	116.00±9.23
**Post 1 week**	0 mm	110.00±9.54	110.05±8.65	110.36±9.73	108.46±8.03
	1.5 mm	112.36±9.31	112.24±9.04	112.42±10.42	111.97±8.49
	−1.5 mm	112.75±9.17	112.00±9.32	113.00±10.81	112.90±8.83
	2.5 mm	117.45±11.21	116.60±10.32	114.96±10.86	114.65±10.59
	−2.5 mm	116.68±11.22	117.05±9.96	115.18±10.67	115.41±11.17
	3.25 mm	116.55±11.74	114.81±10.98	113.49±9.83	114.30±11.13
	−3.25 mm	116.38±12.17	116.69±10.32	113.04±9.01	115.95±10.15
	3.75 mm	115.25±12.55	114.57±10.86	113.09±10.71	114.53±11.18
	−3.75 mm	115.02±13.03	114.98±8.91	112.31±9.26	115.41±10.03
**Post 1 month**	0 mm	107.38±6.86	106.91±5.69	107.60±6.20	107.45±5.83
	1.5 mm	112.25±6.58	111.25±7.41	111.50±6.16	110.94±7.33
	−1.5 mm	110.03±6.98	110.72±7.27	111.00±7.90	111.74±6.58
	2.5 mm	112.94±8.26	112.38±7.13	112.53±6.39	112.48±9.20
	−2.5 mm	113.00±9.14	113.72±6.76	115.40±7.30	113.10±8.31
	3.25 mm	113.91±7.11	111.88±7.41	110.57±7.70	110.61±8.23
	−3.25 mm	113.19±6.89	112.94±7.18	113.50±6.96	112.06±6.85
	3.75 mm	110.50±6.70	110.06±6.67	108.03±8.02	107.94±7.07
	−3.75 mm	109.25±7.43	110.85±6.34	110.53±7.27	109.97±6.70
**Post 6 months**	0 mm	107.78±6.90	108.86±6.26	108.83±6.23	109.50±6.28
	1.5 mm	111.69±8.42	113.25±8.83	110.78±7.71	111.22±8.35
	−1.5 mm	110.69±8.05	110.69±7.36	111.56±6.88	112.19±6.47
	2.5 mm	112.94±9.49	114.58±8.60	113.72±9.11	113.17±7.94
	−2.5 mm	114.56±7.73	114.22±8.91	115.94±7.29	114.53±9.35
	3.25 mm	114.19±8.27	111.94±8.50	111.86±9.59	110.42±8.47
	−3.25 mm	115.00±7.93	115.17±6.18	115.31±8.68	112.50±8.87
	3.75 mm	112.81±8.00	111.31±8.76	110.36±8.29	110.19±9.39
	−3.75 mm	113.11±8.38	114.22±7.22	111.33±6.72	112.89±8.25

+, on the right of the centre;

−, on the left of the centre;

* Statistically significant difference was found between the pair of two points at the same eccentricity. (ANOVA and least squares difference analysis).

### Cap Uniformity

The uniformity of cap thickness was maintained at the different time-points, and the mean cap thickness in the centre was significantly thinner than in the paracentral 1.5 mm to 2.5 mm radius area and peripheral 3.25 mm to 3.75 mm radius area. No statistically significant difference was found between the paracentral and peripheral areas. (Table 2).

**Table 2:** Comparison of the mean cap thickness among the central, paracentral and peripheral area over time (Mean ± SD).

	Central thickness (µm)	Paracentral thickness (µm)	Peripheral thickness (µm)	*P*
Post 1 day	110.81±7.95† ^‡^	115.10±7.82	115.38±7.40	† *P<*0.001^ ‡^ *P<*0.001
Post 1 week	109.58±7.48† ^‡^	113.95±8.69	114.64±9.45	† *P<*0.001^ ‡^ *P<*0.001
Post 1 month	107.32±4.81 † ^‡^	111.94±5.93	110.91±5.14	† *P<*0.001^ ‡^ *P<*0.001
Post 6 months	108.74±5.06† ^‡^	112.86±8.25	112.66±8.33	† *P<*0.001^ ‡^ *P<*0.001

† *vs* Paracentral thickness ^‡^ *vs* Peripheral thickness (a generalized linear mixed-model for repeated measures).

### Refractive Outcomes and their Correlations with Cap Morphology

**Figure 3:**
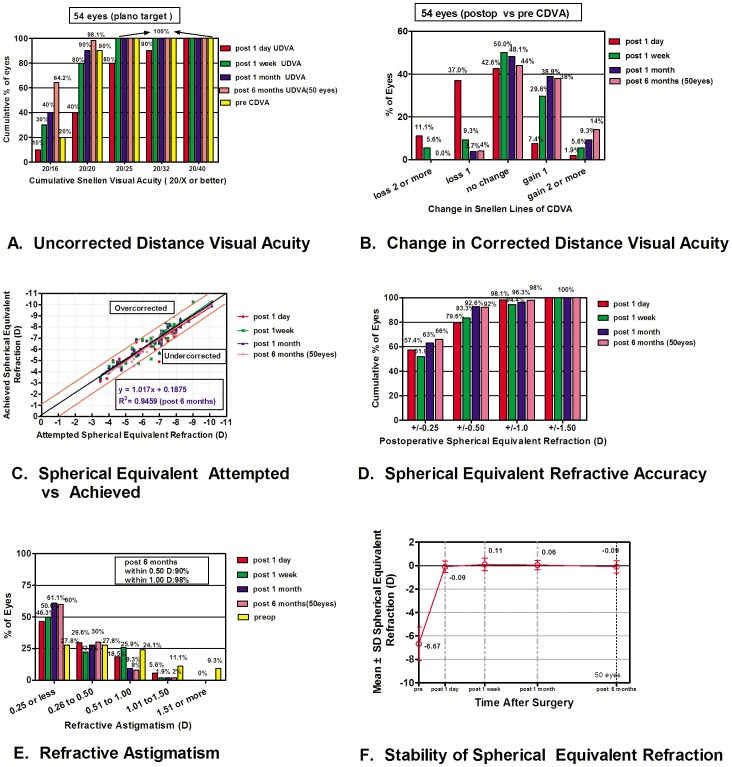
Refractive outcomes after small incision lenticule extraction. UDVA = uncorrected distance visual acuity; CDVA =  corrected distance visual acuity.

The mean absolute values of SE and astigmatism after SMILE were significantly lower than preoperative ones (*P*<.001, Figure 3E, F). Smaller reductions occurred between 1 week and 1 month postoperatively, since when the SE became stabilized (*P* = 1.0, Figure 3F). At the 1 month follow-up, 92.6% of the treated eyes (50/54) fell within ±0.5 D and 100% (54/54) were within ±1.5 D of the intended refractive target (Figure 3D). 50 eyes of 27 patients completed 6 months follow-up, when 98% of the treated eyes (49/50) fell within ±1.0 D. The mean UDVA significantly improved after surgery (*P*<.001), stabilized since 1 month postoperatively. UDVA of 20/25 or better was obtained in 100% of the treated eyes since 1 week after surgery (Figure 3A). CDVA was equal to or better than the preoperative CDVA in 96% of eyes at 6 months postoperatively. Only two eyes lost 1 line of CDVA. (Figure 3B) Spearman correlation analysis revealed a low significant correlation between the central cap thickness and LogMAR UDVA at 1 day (*r_s_* = 0.33, *P = *0.03) and 1 week after surgery (*r_s_* = 0.33, *P* = 0.03), while thinner cap thickness yielded better UDVA. A low significant correlation was also found between index *U* and LogMAR UDVA (*r_s_* = 0.34, p<0.05) and between index *U* and LogMAR CDVA (*r_s_* = 0.32, p<0.05) after 1 week. (Table 3).

**Table 3:** Refractive outcomes over time (Mean ± SD) and correlation with cap morphology.

Parameters	Post 1 day			Post 1 week			Post 1 month			Post 6 months		
	Correlation *P (r_s_)*			Correlation *P (r_s_)*			Correlation *P (r_s_)*			Correlation *P (r_s_)*		
		CcT	*U*		CcT	*U*		CcT	*U*		CcT	*U*
SE (D)	−0.09±0.49	0.26	0.33	0.11±0.53	0.34	0.06	0.06±0.40	0.48	0.68	−0.09±0.52	0.71	0.72
LogMAR UDVA	0.08±0.11	0.03*(0.33)	0.60	−0.01±0.08	0.03*(0.33)	0.02*(0.34)	−0.02±0.07	0.47	0.72	−0.08±0.07	0.37	0.08
LogMAR CDVA	0.03±0.09	0.30	0.93	−0.03±0.07	0.07	0.03*(0.32)	−0.06±0.07	0.10	0.89	−0.08±0.07	0.07	0.91
Astigmatism (D)	−0.44±0.40	0.21	0.10	−0.38±0.34	0.48	0.42	−0.32±0.31	0.44	0.39	−0.34±0.36	0.79	0.71

SE = spherical equivalent; UDVA = uncorrected distance visual acuity; CDVA =  corrected distance visual acuity; CcT = central cap thickness; *U* =  a gross index of uniformity, the standard deviation of the cap thickness of total 33 points;

* Statistically significant correlation was found; *r_s_ = *Spearman correlation coefficient.

## Discussion

SMILE is an advanced form of ReLEx because it requires no retractable flap. It has been proven to be an effective and safe procedure both in our study and some earlier reports [1]–[6]. The present study demonstrated excellent predictability for SMILE both in central cap thickness and diameter. Similar to previous FS-LASIK flaps studies [7], [13]–[15], the VisuMax FS yielded SMILE caps 10.81 µm, 9.58 µm, 7.32 µm and 8.74 µm thicker than the intended thickness at 1 day, 1 week, 1 month and 6 months respectively after surgery. In addition, the reproducibility of the cap thicknesses was comparable to the values published by commercially available FS systems [7], [9], [14], [15], showing the smallest SD of 4.81 µm at 1 month postoperatively. The mean achieved cap diameter in our study was 7.61±0.07 mm, 7.57±0.06 mm on the 0° and 90° meridians at 1 month postoperatively, with higher accuracy and reproducibility than the previous studies. Holzer et al. [16] demonstrated that the measured flap diameter had a median difference between 0% and 3.16% or ±0.3 mm from the intended diameter. Similar findings were also reported by Binder [17], who found a range differing up to 0.6 mm from the intended flap diameter and a SD between 0.12 and 0.26 mm. The discrepancy between our study and others may result from the differences of laser parameters among the various FS systems, or because of the different measurement methods. Binder [18] suggested that the speed of the FS was important and correlated with flap thickness predictability, and he also reported that the FS photodisrupted more deeply when set between 110 µm and 120 µm with a SD of 12 µm but dissected less deeply when set between 130 µm and 140 µm with a SD of 18.5 µm [17]. Last but not least, the time taken between the femtosecond cuts for the refractive lenticule creation may affect the final shape of the lenticule [2]. The new generation of VisuMax FS (higher pulse frequency and lower pulse energy) applied in our study was proposed to have improved the accuracy and quality of lenticule creation. Greater speed has enabled decreased pulse energy, resulting in smaller photodisruption spot diameter. In this regard, smaller spot diameter adds more precision in the dissection plane and therefore yields greater accuracy in the depth of the lenticule cut. Nevertheless, further investigation is warranted to evaluate the various factors that may influence the cap creation. In addition, our data also suggested the horizontal diameter was significantly larger than the vertical diameter, and we suspected this may be related to the superiorly positioned small incision and the manual operation during the lenticule separation procedure. The clinical impact of this phenomenon requires further investigation.

In the current study, we found that the cap thickness was greatest initially from 1 day to 1 week postoperatively due to possible corneal swelling, which decreased over the following 1 month as the edema subsided and stabilized till 6 months postoperatively, consistent with the trend in refractive recovery in the current study and previous clinical research [3]. In contrast, it was reported that LASIK surgeries were associated with corneal swelling and recovery during the first postoperative week and thickening after the first month [19], [20]. A few factors might explain this discrepancy. Firstly, several processes occur during the early postoperative period: resorption of fluid induced by intraoperative irrigation, biomechanical hydration shift, epithelial thickness modulation in response to wound healing and interface reflectivity change [19]. These changes may have complex effects on flap or cap thickness and account for the difference. Secondly, the diverse wound healing response and inflammatory infiltration after the two distinct procedures may also contribute. Riau et al. [21] implied that the excimer laser treatment in LASIK stimulated a higher degree of inflammation in comparison with ReLEx, by releasing more cytokines and chemokines that recruit the inflammatory cells to the injury site. They demonstrated that as an advanced ReLEx technique, SMILE has a much reduced incision size and its postoperative inflammatory infiltration may be reduced further due to less epithelial disruption [21]. Thirdly, the extent of the inflammatory response was also dependent on the pulse frequency and pulse energy delivered by the FS. However, the mechanism of the impact of cap creation and corneal wound healing on cap thickness after SMILE still remains unclear. Further studies incorporating other instruments such as confocal microscopy would be needed to confirm our hypothesis.

In general, our results showed good regularity of cap thickness with no difference among the different measurement points at each eccentricity in the early postoperative period. Regularity in flap or cap thickness is important in its role in providing adequate biomechanical support and strength as well as in producing successful refractive and wavefront outcomes [22], [23]. Interestingly, we found that two pairs of points in 1.5 mm and 2.5 mm eccentricities had significant difference in cap thickness only at 1 day postoperatively, which might have an impact on visual outcomes as both postoperative UDVA and CDVA were worse at the first day than other time-points after surgery (Figure 3A, B). Some of the latest studies [24], [25] on the evaluation of the lenticule surface morphology could explain this phenomenon. Three different types of irregularity on the lenticule surface were revealed [24], [25]: 1) Tissue bridges, which were embedded in areas of cavitation bubbles. This could be described as residual fibers between flap and lenticule interface after bubble formation has been completed. 2) Crater-like cavities along the cutting surface. These were attributed to gas bubbles and the craters became bigger as single bubbles merged. 3) Grooves ran criss-cross over the cutting surface, which was assumed to be related to surgical manipulation, though they did not affect the surface regularity as much as the other two types of irregularities. We supposed that the regularity improved after clearance of the opaque bubble layer and with tissue healing over time.

With regard to the uniformity, our findings indicated that the corneal caps may be thinner centrally than in the periphery, but the difference in thickness between the paracentral and peripheral areas was not statistically significant, showing a general uniform morphology of the caps at the different time-points. A uniform cap may contribute to the predictable refractive results with regard to the SE and the stability of the achieved refractive change in the present study. These results were similar to those in FS flaps studies. Kim et al. [26] found that the mean achieved flap thickness in the central 1.5 mm radius area was thinner than that in the peripheral 3.0 to 4.0 mm radius area, and the difference ranged from 3.33 µm to 5.58 µm. It was reported in other studies that the mean FS flap thickness increased slightly toward the periphery [27]. One of the possible reasons may be due to the biomechanical characteristics of the cornea. Dawson et al. [28] demonstrated a difference in tensile strength between the central and peripheral cornea, with increasing tensile strength moving from the center to the periphery. The VisuMax system maintains a curved corneal surface, although this approach may be advantageous for procedures requiring intrastromal beam localization under natural corneal curvature, it may contribute to center–peripheral disparity [29]. In addition, the decreasing incidence angle of the OCT beam might increase geometric and refractive distortions that may also be responsible for this phenomena [30]. However, we speculate that such difference might be simply necessary for the creation of a convex lenticule. To create a convex lenticule we might have to design an elevated anterior surface (protrusion in the center), leaving the cap a thinner-center & thicker-periphery pattern. Moreover, a comparison of currently available FS systems showed that the development of cavitations was reduced with systems using higher frequencies and lower pulse energies [31], which may support our speculation that the new generation of VisuMax system in the present study might provide an even better dissection plane and help improve the regularity and uniformity of the cap creation of SMILE.

Furthermore, we found that the postoperative UDVA was associated with the central cap thickness at 1 day and 1 week after SMILE. This may be attributed to corneal edema, which led to the thickened central cap thickness and affected the visual acuity the most at that time. As the edema usually resolved within 1 month, the impact of cap thickness on visual acuity faded away accordingly. In addition, a low correlation was detected between the uniformity index and both UDVA and CDVA at 1 week postoperatively. We hypothesize that the reason why the effect of cap uniformity on refractive outcomes was not obvious at other time-points was because the corneal swelling played a major role in visual acuity at 1 day while other more significant factors were manifest at 1 month and 6 months follow-up. Nevertheless, with the week correlations observed in the present study, the impact of corneal cap morphology on the early refractive outcomes may be mild and thus of minimal clinical significance. Further studies should focus on the long-term effect of SMILE on visual quality consisting of higher order corneal aberrations, contrast sensitivity, etc.

In conclusion, the present study demonstrated that the corneal cap creation in SMILE with the advanced generation of VisuMax FS system was predictable with good reproducibility, regularity and uniform morphology. The cap morphology might have a mild effect on refractive outcomes in the early stage. Further studies are warranted to address the clinical impact of cap morphology on visual quality.
